# *Oryza sativa* Chloroplast Signal Recognition Particle 43 (OscpSRP43) Is Required for Chloroplast Development and Photosynthesis

**DOI:** 10.1371/journal.pone.0143249

**Published:** 2015-11-23

**Authors:** Xiang-guang Lv, Yong-feng Shi, Xia Xu, Yan-lin Wei, Hui-mei Wang, Xiao-bo Zhang, Jian-li Wu

**Affiliations:** State Key Laboratory of Rice Biology, China National Rice Research Institute, Hangzhou, Zhejiang, People’s Republic of China; University of Western Sydney, AUSTRALIA

## Abstract

A rice chlorophyll-deficient mutant w67 was isolated from an ethyl methane sulfonate (EMS)–induced IR64 (*Oryza sativa* L. ssp. *indica*) mutant bank. The mutant exhibited a distinct yellow-green leaf phenotype in the whole plant growth duration with significantly reduced levels of chlorophyll and carotenoid, impaired chloroplast development and lowered capacity of photosynthesis compared with the wild-type IR64. Expression of a number of genes associated with chlorophyll metabolism, chloroplast biogenesis and photosynthesis was significantly altered in the mutant. Genetic analysis indicated that the yellow-green phenotype was controlled by a single recessive nuclear gene located on the short arm of chromosome 3. Using map-based strategy, the mutation was isolated and predicted to encode a chloroplast signal recognition particle 43 KD protein (cpSRP43) with 388 amino acid residuals. A single base substitution from A to T at position 160 resulted in a premature stop codon. *OscpSRP43* was constitutively expressed in various organs with the highest level in the leaf. Functional complementation could rescue the mutant phenotype and subcellular localization showed that the cpSRP43:GFP fusion protein was targeted to the chloroplast. The data suggested that *Oryza sativa* cpSRP43 (OscpSRP43) was required for the normal development of chloroplasts and photosynthesis in rice.

## Introduction

Chlorophyll is one of the most abundant biological molecules on earth and plays an essential role in photosynthesis, yet the regulation of chlorophyll metabolism remains incompletely understood [1]. Chlorophyll-deficient phenotypes are usually caused by mutations of genes associated with chlorophyll metabolism or chloroplast biogenesis, thus chlorophyll-deficient mutants are regarded as the ideal material for the understanding of chlorophyll metabolism, chloroplast biogenesis and photosynthesis. Up to now, chlorophyll-deficient mutants have been reported in many plant species including *Arabidopsis*, rice, maize and tomato [2–5]. In *Arabidopsis*, chlorophyll metabolism has been extensively studied and 27 genes encoding for 15 enzymes have been identified and well characterized [6].

Partially or fully loss of function to the components in chlorophyll biosynthesis could lead to abnormal phenotypes. For instance, the initial step for tetrapyrrole formation is catalyzed by a NADPH-dependent glutamyl-tRNA reductase (GluTR) encoded by the nuclear *HEMA* gene [7]. The transgenic *Arabidopsis* plants expressing antisense *HEMA1* mRNA exhibit varying degrees of chlorophyll deficient phenotype, ranging from patchy yellow to complete yellow [8]. In addition, the 3,8-divinyl protochlorophyllide a-8-vinyl reductase (DVR) is indispensable for monovinyl chlorophyll biosynthesis [9]. A point mutation of *DVR* gene can lead to a pale green phenotype in *Arabidopsis* [10] while a nine-nucleotides deletion of *DVR* gene can cause the yellow-green leaf phenotype in rice [11]. Furthermore, loss of function in Mg-cheletase, chlorophyll synthase and chlorophyllide oxygenase could all result in different leaf color variation in rice [5,12,13].

Similarly, loss of function to the enzymes participating in chlorophyll breakdown pathway would also result in leaf phenotypic variation [14, 15]. For example, chlorophyll b reductase, responsible for the conversion of chlorophyll b to chlorophyll a, is encoded by two genes, *NON-YELLOW COLORING 1* (*NYC1*) and *NYC1-like* (*NOL*) [16, 17]. Impaired function of either *NYC1* or *NOL* would result in a non-functional stay-green phenotype in rice [18, 19].

The normal development of chloroplasts is necessary for the regulation of chlorophyll metabolism and thus associated with the leaf color variation. A chloroplast is estimated to contain several thousands of proteins encoded mainly by the nuclear genes [20]. Defects in these genes would result in impaired development of chloroplasts and changes of leaf color phenotype. For example, the defect of Toc159 protein, an important component of the receptor complex located in both the cytosol and the outer envelope membrane [21, 22], causes a non-photosynthetic albino phenotype in *Arabidopsi*s mutant *ppi2* [23]. The impaired function of VIPP1 (vesicle-inducing protein in plastids 1) results in a pale-green phenotype in *Arabidopsis hcf155* mutant at the early developmental stage [24, 25]. Furthermore, the disruption of the *Thf1* (*Thylakoid formation 1*) gene via T-DNA insertion leads to the impaired thylakoid formation and variegated leaves in *Arabidopsis* [26].

In this study, we identified a rice chlorophyll-deficient mutant w67, which exhibited distinct yellow-green leaves with reduced levels of photosynthetic pigments, abnormal chloroplast development and impaired photosynthesis compared with the wild type. The mutant phenotype was controlled by a single recessive nuclear gene. Using map-based strategy, we show that a single base substitution in the *chloroplast-targeted signal recognition particle 43* (*cpSRP43*) gene was responsible for the yellow-green phenotype. Our results show that the *Oryza sativa* cpSRP43 (OscpSRP43) is necessary for the normal development of chloroplast and photosynthesis in rice.

## Materials and Methods

### Plant materials

The yellow green mutant w67 (originally coded as E17707-7) was derived from IR64 (*Oryza sativa* L. ssp. *indica*) mature seed treated with ethyl methane sulfonate (EMS) at the International Rice Research Institute [27]. The yellow-green leaf phenotype has been stably inherited for more than nine generations under the field and greenhouse conditions in Hangzhou, Zhejiang, China. The mutant and wild-type IR64 were grown in the paddy field at the China National Rice Research Institute (CNRRI) for evaluation of main agronomic traits including plant height, tiller number, panicle length, and 1,000-grain weight during the rice growing season in 2013. Means from three replications were used for analysis.

### Measurement of photosynthetic pigments and chlorophyll fluorescence

The contents of chlorophyll (Chl) and Carotenoid (Car) were measured using a UV/VIS spectrophotometer according to the method of Arnon with minor modifications [28]. 0.1–0.2 g fresh second upper leaves were taken in triplicates from 8-, 10-, 12- and 14-week-old plants, and then marinated in 95% ethanol in the dark at 4°C for 48 h. After a brief spin, the supernatants were analyzed with a lambda 25 UV/VIS Spectrophotometer (Perkin Elmer, USA) at 665, 649 and 470 nm, respectively. The means from three replications were used for analysis.

Chlorophyll fluorescent parameters of w67 and IR64 were measured on the 11-week-old w67 and IR64 plants at 9:00–10:00 am under field conditions following the method described by Huang et al [29].

### Transmission electron microscopy

Leaf samples of 4-week-old w67 and IR64 were fixed in 2.5% (v/v) glutaraldehyde (0.2 M phosphate buffer, pH 7.2) at 4°C for 16 h. After separately rinsed with phosphate buffer for three times, the samples were treated with 1% (w/v) osmium tetroxide at 4°C for 4 h, and washed with phosphate buffer again. Then, the samples were dehydrated through ethanol series [50%, 70%, 85%, 95%, 100% (v/v)]. Ethanol was subsequently replaced by a series of Spurr’s resin dilutions [25%, 50%, 75%, 100% (v/v)]. At last, the samples were embedded in Spurr’s resin at 65°C for 16 h to thin sectioning. Sections were stained with uranyl acetate and observed by a Tecnai G^2^ F20 S-TWIN transmission electron microscope at the College of Agriculture and Biotechnology, Zhejiang University.

### Map-based cloning of *w67*

For genetic analysis, we crossed w67 with Moroberekan, R9308 and 02428 in Hangzhou Experimental Station at CNRRI in 2011, respectively. F_1_ plants were grown at the Lingshui Experimental Station in 2012 and selfed to generate F_2_ populations. F_2_ individuals from all three crosses were grown in Hangzhou in the same year and phenotyped for segregation analysis. Three segregation lines each from w67/Moroberekan and w67/02428 were phenotyped for confirmation of the segregation the next season in Hangzhou in 2013.

The F_2_ population derived from w67/Moroberekan was chosen for mapping. Bulked segregant analysis was first used for preliminary mapping of the mutation. Equal amount of leaf blades from each of 10 wild-type and 10 mutant type plants were collected for DNA extraction to form a wild-type DNA pool and a mutant-type DNA pool, respectively. To fine-map the *w67* gene, a total of 801 F_2_ mutant-type individuals were genotyped. DNA of parents and F_2_ individuals was extracted following the mini-preparation method [30]. Simple sequence repeat (SSR) markers were obtained from the website (http://www.gramene.org/) while insertion/deletion (InDel) markers were designed using the Primer 5.0 and DNAStar 5.0 software after comparison of the sequences between the japonica cultivar Nipponbare and the indica cultivar 9311 in the public databases: RGP (http://rgp.dna.affrc.go.jp/E/toppage.html), Gramene (http://gramene.org/genome_browser/index.html) and the Gene Research Center of the Chinese Academy of Sciences (http://rice.genomics.org.cn/rice/index2.jsp). The primers were synthesized by Sangon Biotech Co. Ltd (Shanghai, China) and listed in S2 Table. PCR reaction and detection were carried out as described previously [31].

### Genetic complementation assay

For complementation of the mutant phenotype, a 6.2 kb wild type genomic fragment covering a 4.1 kb upstream sequence from the start codon, 1.3 kb from the start to the stop codon and a 0.8 kb downstream sequence from the stop codon was amplified by PCR using the ComF/R primers (ComF, 5′- GCTCTAGAGCGTGCTAAGGAGCCTCTTGAAT-3′ and ComR, 5′- CACTGCAGTGGCTTCACTGCCAAAACGAG-3′). The PCR product was double-digested with *Xba* I and *Pst* I, and the fragment was recovered using an Axygen DNA gel extraction kit. Then, the fragment was inserted into the plant expression vector pCAMBIA1300 with a hygromycin-resistant gene to generate a new construct designated as pCAMBIA1300-w67 (S1A Fig) which was transformed into the embryogenic calli induced from mature seeds of w67 according to the *Agrobacterium tumefaciens*-mediated transformation method [32]. Transformants were confirmed for the presence of the transgene by PCR using the P67F/R primers (P67F, 5′-TGGAGGCTGTCCTACGACACC-3′, P67R, 5′-GACGAGGTACTCCGTGGCG-3′).

### Subcellular localization

The coding sequence of *OscpSRP43* was amplified using the specific primers SLW67 F/R (SLW67F, 5’-AGTCCGGAGCTAGCTCTAGAATGGAGGCTGTCCTACGACACCCA-3’; SLW67R, 5’-GCTCACCATGGATCCCCCGGGCCCGGCGACCGGCGGCGG-3’) The PCR product was inserted to the 5’-terminal of *GFP* driven by the CaMV 35S promoter in the transient expression vector PAN580 to form a new construct PAN580-w67 (S1B Fig) which was then introduced into the rice protoplasts according to the protocol described previously [33]. The GFP fluorescence was observed by a Leica TCS SP5 confocal laser scanning microscope.

### Quantitative reverse transcription PCR (qRT-PCR)

To determine the expression profile of the *OscpSRP43*, total RNA was extracted from the organs including roots, stems, leaves, leaf sheaths and panicles at the heading stage of IR64 using the TRIzol method following the manufacture’s instruction (Aidlab biotechnologies CO. Ltd, China). For qRT-PCR analysis of genes associated with Chl biosynthesis and photosynthesis (S3 Table), total rice RNA was extracted from the leaves of w67 and IR64 at the tillering stage following the method mentioned above. The first-strand cDNA was synthesized using First Strand Cdna Synthesis Kit according to the manufacturer’s protocol [TOYOBO Biotech (Shanghai) CO. LTD, Japan]. qRT-PCR was performed on a Thermal Cycler DiceTM Real Time System II following the manufacturer’s instruction [TaKaRa Biotechnology (Dalian) CO. LTD, Japan]. The 2–ΔΔCT method was used to analyze the relative transcript levels in gene expression with the means from three replications.

### Sequence alignment and phylogenetic analysis

BLAST analysis was performed on the NCBI website (http://www.ncbi.nlm.nih.gov/) to search for homologs of OscpSRP43. A total of 8 sequences from 8 species were identified. The sequences were aligned using BioEdit software and the neighbor-joining tree was generated using the Poisson correction method in MEGA 5.1 software. Bootstrap replication with 1000 times was used for a statistical support for the nodes in the phylogenetic tree.

## Results

### Phenotype of w67 mutant

**Fig 1:**
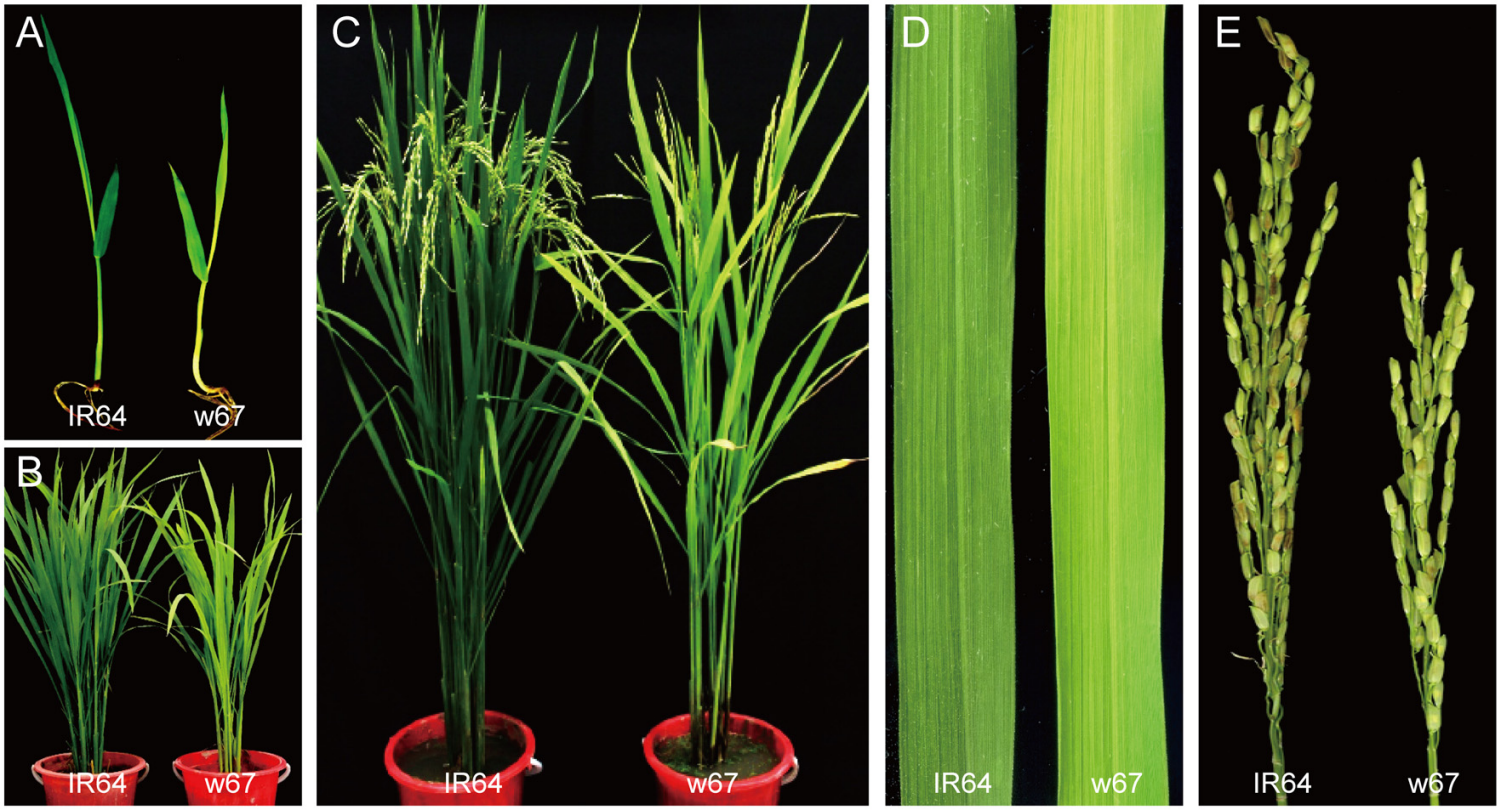
Phenotype of w67 mutant. A-C, phenotype of IR64 and w67, 5-day-old (A), 9-week-old (B) and 14-week-old (C); D, Leaf blade of 4-week-old IR64 and w67; E, panicles of IR64 and w67.

Under the field and greenhouse conditions in Hangzhou, China, the mutant displayed a distinct yellow-green leaf phenotype in the whole life period, and exhibited a slightly retarded growth compared with the wild-type IR64 (Fig 1A–1D). In contrast to the wild type, the main agronomic traits including plant height, tillering number/plant, length of panicle and grain number per panicle were remarkably reduced in the mutant (Fig 1E and Table 1). Furthermore, the heading date of w67 was about 10 d later than that of the wild type.

**Table 1:** The main agronomic traits of w67 and wild type IR64.

Material	Tillering number	Plant height (cm)	Panicle length (cm)	No. filled grains/ panicle	1,000-grain weight (g)
w67	12.1 ± 1.2**	91.27 ± 3.41**	23.50 ± 1.32**	86.67 ± 9.07**	25.34 ± 0.46
IR64	16.3 ± 1.6	118.50 ± 2.64	27.37 ± 0.32	118 ± 6.56	24.89 ± 0.34

Values are means ± SD; n = 3;

**, Highly significance at P ≤ 0.01.

### w67 is a chlorophyll-deficient mutant

The contents of chlorophyll a (Chl a), chlorophyll b (Chl b), and carotenoids (Car) in w67 and wild-type plants were measured at four different developmental stages. The results indicated that the contents of Chl a, Chl b and Cars in the w67 mutant were significantly lower than those of the wild-type IR64 in all developmental stages (Table 2). Reduction of Chl a, Chl b and Car in the mutant ranged from 45.56–60.71%, 29.25–45.71% and 65.63–83.87% respectively compared with the wild type IR64. However, the Chla/b ratios appeared significantly higher in the mutant than those of the wild type at all the stages (Table 2). These results indicate that w67 was a photosynthetic pigments-deficient mutant in the whole life duration with a severer declination in Chl b level.

**Table 2:** The pigment contents in w67 and wild-type IR64 (mg/g fresh weight).

Stage	Material	Chl a	Chl b	Car	Total Chl	Chl a/b
8-week-old	w67	1.31±0.12**	0.31±0.02**	0.52±0.04**	1.63±0.14**	4.24±0.18**
	IR64	2.94±0.11	1.06±0.11	0.62±0.01	4.00±0.21	2.79±0.18
10-week-old	w67	1.36±0.01**	0.32±0.02**	0.38±0.01*	1.68±0.02**	4.29±0.33**
	IR64	2.24±0.40	0.70±0.14	0.48±0.09	2.95±0.54	3.20±0.06
12-week-old	w67	1.43±0.05**	0.4±0.01**	0.21±0.03**	1.83±0.06**	3.58±0.07**
	IR64	2.48±0.19	0.96±0.11	0.30±0.02	3.45±0.30	2.59±0.11
14-week-old	w67	1.31±0.04**	0.39±0.02**	0.21±0.02**	1.71±0.05**	3.33±0.06**
	IR64	2.59±0.04	1.08±0.04	0.32±0.02	3.68±0.08	2.40±0.06

The values indicate the mean ± SD from three independent determinations,

*, Significance at P ≤0.05;

**, Highly significance at P ≤ 0.01.

Chl, Chlorophyll; Car, Carotenoid.

### Chloroplast development and photosynthesis were impaired in w67

**Fig 2:**
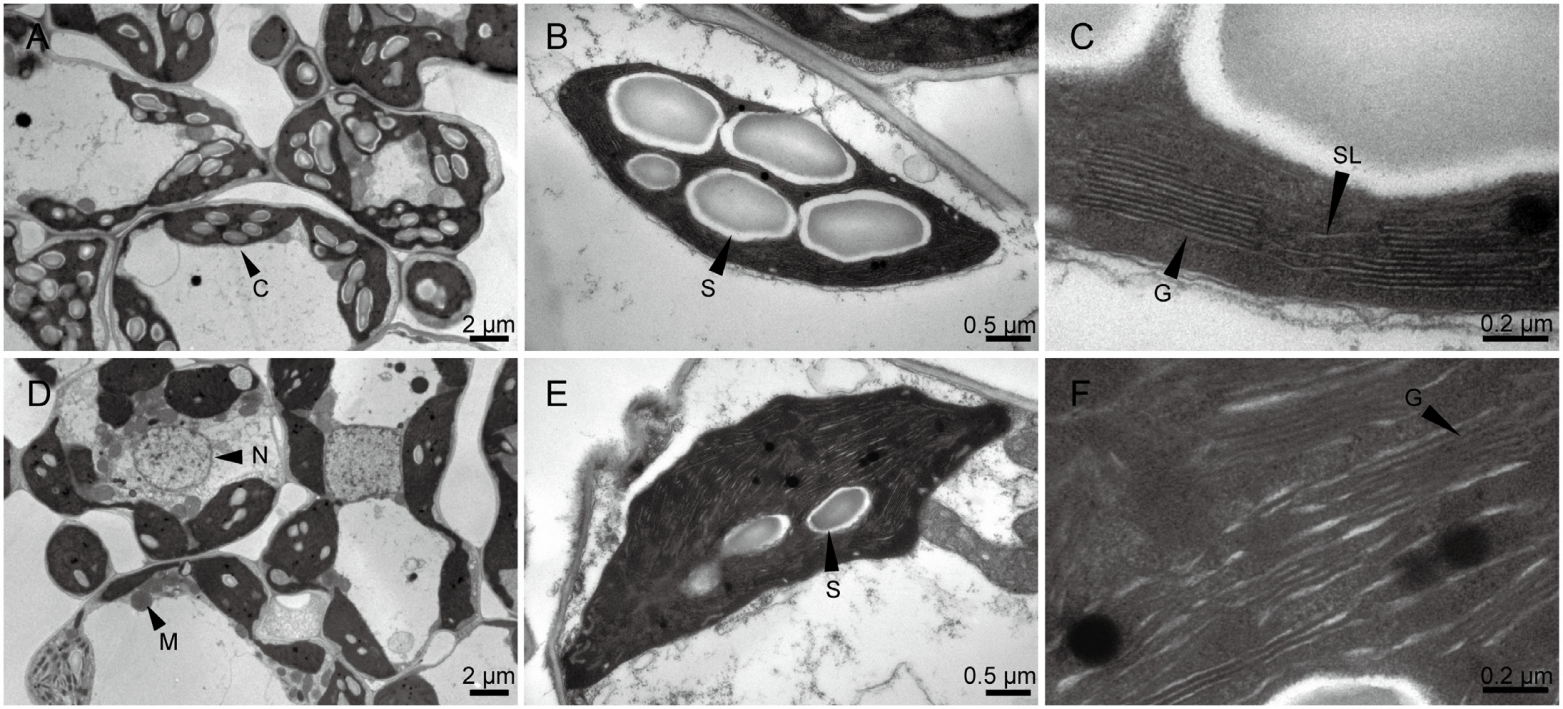
Chloroplast ultrastructure of IR64 and w67. **A-C,** 4-week-old IR64; D-F, 4-week old w67; C chloroplast, M mitochondria, N nucleus, S starch granule, G granum, SL stroma lamella.

To investigate the chloroplast development in the w67 mutant, we compared the ultrastructure of chloroplasts in the second upper leaves from the 4-week-old mutant and wild-type plants using transmission electron microscopy (TEM). The results showed that fully developed chloroplasts in mesophyll cells were presented in the leaves of the wild type IR64 (Fig 2A–2C) while reduced number of starch granules, decreased number of thylakoid lamellar layers, poor arrangement of grana and increased number of osmiophilic granules were observed in the mutant leaves (Fig 2D–2F), indicating that the mutation significantly affected the development of chloroplasts.

Chlorophyll fluorescence is a non-invasive measurement of photosystem II (PSII) activity. We examined and compared chlorophyll fluorescent parameters between the 11-week-old mutant and wild type IR64. As shown in Table 3, the maximal chlorophyll fluorescence yield (Fm), the maximum photochemical quantum yield of PS II (Fv/ Fm), the effective photochemical quantum yield of PS II (ΦPSII) and the rate of relative electron-transport rate (ETR) were significantly decreased in the mutant compared with the wild type. The results suggested that the function of photosystem II was impaired in the mutant.

**Table 3:** Chlorophyll fluorescence parameters in the 11-week-old w67 mutant and wild-type IR64.

Material	Fo	Fm	Fv/Fm	ΦPSII	ETR (μmol electron m^−2^s^−1^)
w67	0.16 ± 0.00	0.51 ± 0.04**	0.68 ± 0.03**	0.36 ± 0.03**	177.67 ± 9.02**
IR64	0.17 ± 0.01	0.82 ± 0.02	0.79 ± 0.01	0.48 ± 0.03	243.33± 11.68

Fo, minimum fluorescence; Fm, maximum fluorescence; Fv/Fm, maximum photochemical quantum yield of PSII (photosystem II); ΦPSII, effective photochemical quantum yield of PSII. The means from three measurements were used for analysis.

**, Highly significance at P ≤ 0.01

### Map-based cloning of the *w67* gene

All F_1_ plants generated from the crosses of w67/02428, w67/Moroberekan and w67/R9308 displayed normal green leaves as that of IR64, indicating that the yellow-green phenotype was controlled by a recessive gene (s). In all three F_2_ populations, no inter-mediate leaf type plants were found, and the number of normal-green leaf plants and yellow-green leaf plants fitted to the 3:1 ratio (Table 4). To further confirm this observation, 3 segregating F_3_ lines each derived from both crosses w67/02428 and w67/Moroberekan were planted and phenotyped, again they all fitted to the 3:1 segregation ratios (S1 Table). Taken together, the data indicated that the yellow-green leaf phenotype was controlled by a single recessive nuclear gene.

**Table 4:** Genetic analysis of the yellow-green phenotype in w67.

Cross	F_1_	F_2_ individual		χ^2^_(3:1)_
		No. green plant	No. yellow- green plant	
w67 / 02428	Green leaf	611	209	0.104
w67/Moroberekan	Green leaf	399	129	0.090
w67/ R9308	Green leaf	781	253	0.156

**Fig 3:**
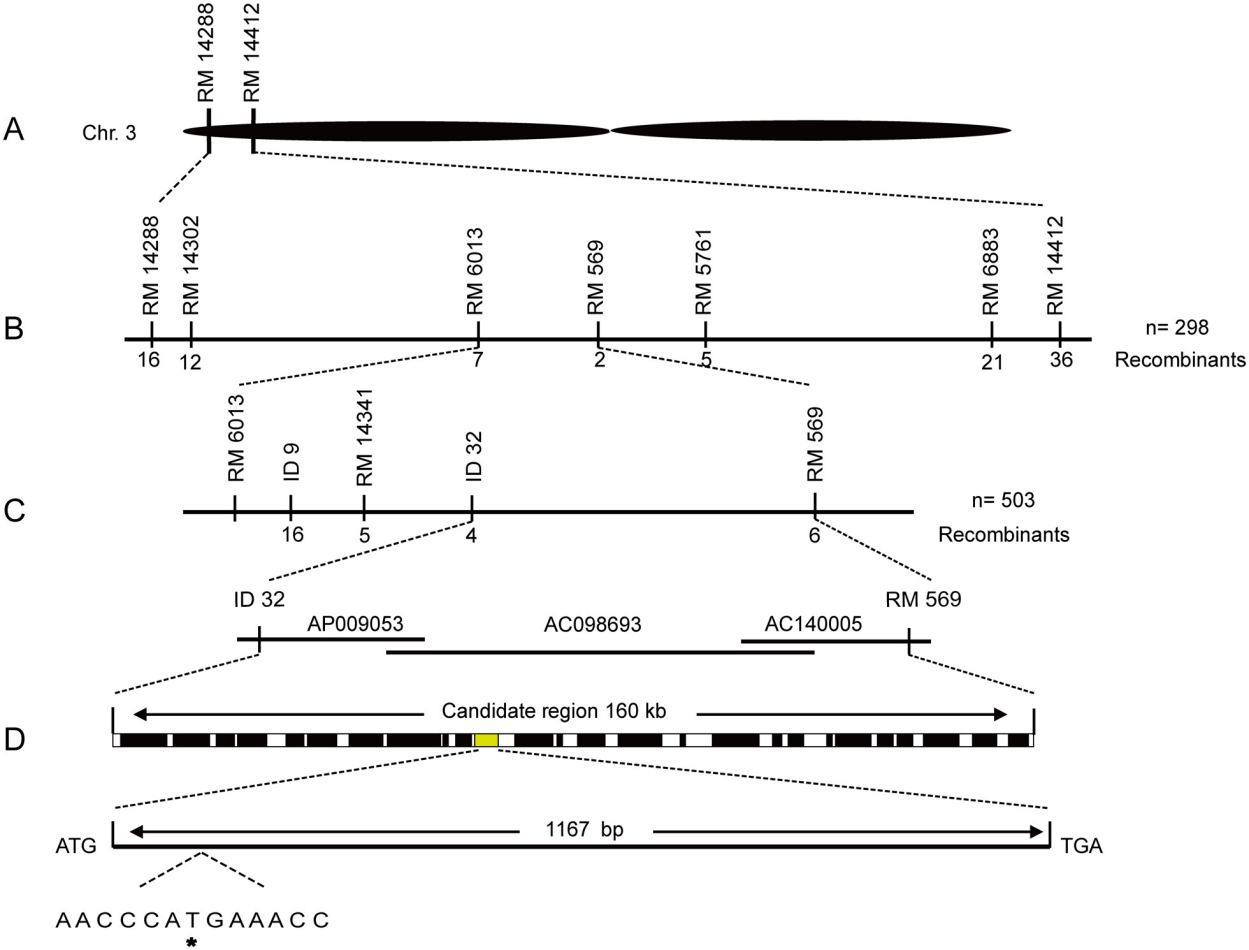
Map-based cloning of *w67*. A, The *w67* locus was mapped to short arm of chromosome 3 between SSR markers RM14288 and RM14412; B, The w67 locus was narrowed down to about 274-kb region between RM6013 and RM569 using 298 F_2_ mutant individuals from cross of w67/R9308; C, The w67 locus was further mapped to about 160-kb region covering three BAC clone AP009053, AC098693 and AC140005 between InDel maker ID32 and RM569 using 503 F2 mutant type plants from cross of w67/Moroberekan; D, 26 ORFs were predicted in the mapped region and a single nucleotide substitution from A to T at position 130 in *LOC_Os03g03990* was identified. Markers used for mapping are listed in S2 Table.

To isolate this recessive gene, tentatively termed as *w67*, we performed map-based cloning using F_2_ populations derived from w67/R9308 and w67/Moroberekan. Initial mapping based on 72 mutant-type individuals revealed that the *w67* gene was located on the short arm of rice chromosome 3 between the SSR makers RM14288 and RM14412 (Fig 3A). Then we genotyped 298 F_2_ mutant individuals derived from w67/R9308 and delimited the mutation to a 274 kb region (Fig 3B). To further refine its position, we genotyped 503 F_2_ mutant-type plants derived from w67/Moroberekan and delimited *w67* to a 160 kb region flanked by ID32 and RM569 covering three BAC clones AP000615, AC098693 and AC140005 (Fig 3C). A total of 26 open reading frames (ORFs) were annotated in the Rice Genome Annotation Project (http://rice.plantbiology.msu.edu/) database. Sequence alignments between the wild-type and mutant alleles revealed that a single-nucleotide substitution from A to T at position 160 in *LOC_Os03g03990* leading to a premature stop codon in the mutant was identified (Fig 3D). This point mutation presumably generated a truncated protein containing only 53 amino acid residues and lacked all the known domains.

**Fig 4:**
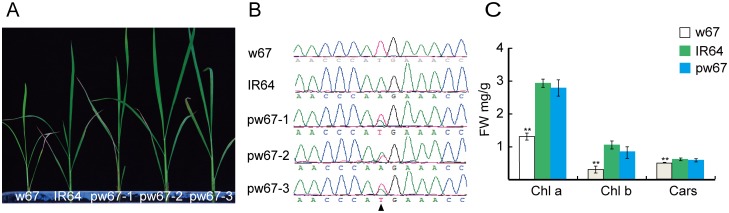
Functional complementation at the *w67* allele. A, Phenotypes of w67, IR64 (WT) and complemented plants (pw67-1, pw67-2 and pw67-3); B, Nucleotides at the mutation sites in w67, IR64 and complemented plants; C, Levels of Chl a, Chl b and Cars in w67, IR64 and complemented plants at 8-week old. **, Highly significance at P ≤ 0.01.

Therefore, we assumed *LOC_Os03g03990* was the candidate gene responsible for the yellow-green phenotype. To test, we constructed a complementary vector pCAMBIA1300-w67 harboring the wild-type allele, and introduced it into the mature-embryo induced calli from w67 via *Agrobacterium*-mediated transformation. A total of 31 independent transformants were obtained, 22 out of them exhibited a normal-green leaf phenotype similar to the wild-type IR64 (Fig 4A). PCR analysis indicated that all 22 complemented transgenic plants contained the transgene (Fig 4B). In addition, the contents of Chl a, Chl b and Car in the 8-week-old transgenic lines were similar to the levels of the wild-type IR64 (Fig 4C). Taken together, the data demonstrated that the single-nucleotide substitution in *LOC_Os03g03990* gene was responsible for the yellow-green leaf phenotype in the w67 mutant.

### W67 is a homolog of chloroplast signal recognition particle 43 (cpSRP43)

Sequence analysis using the public database (http://www.gramene.org) reveals that *w67* is highly homologous to the *Arabidopsis CAO* gene encoding the chloroplast signal recognition particle 43 protein (AtcpSRP43). Therefore, we term the *w67* mutation as *Oryza sativa cpSRP43* (*OscpSRP43*). *OscpSRP43* is a single copy gene in the rice genome and does not contain any intron. It putatively encodes a polypeptide consisting of 388 amino acid residues with a calculated molecular mass of approximately 42.1 kD and an isoelectric point of 4.4.

**Fig 5:**
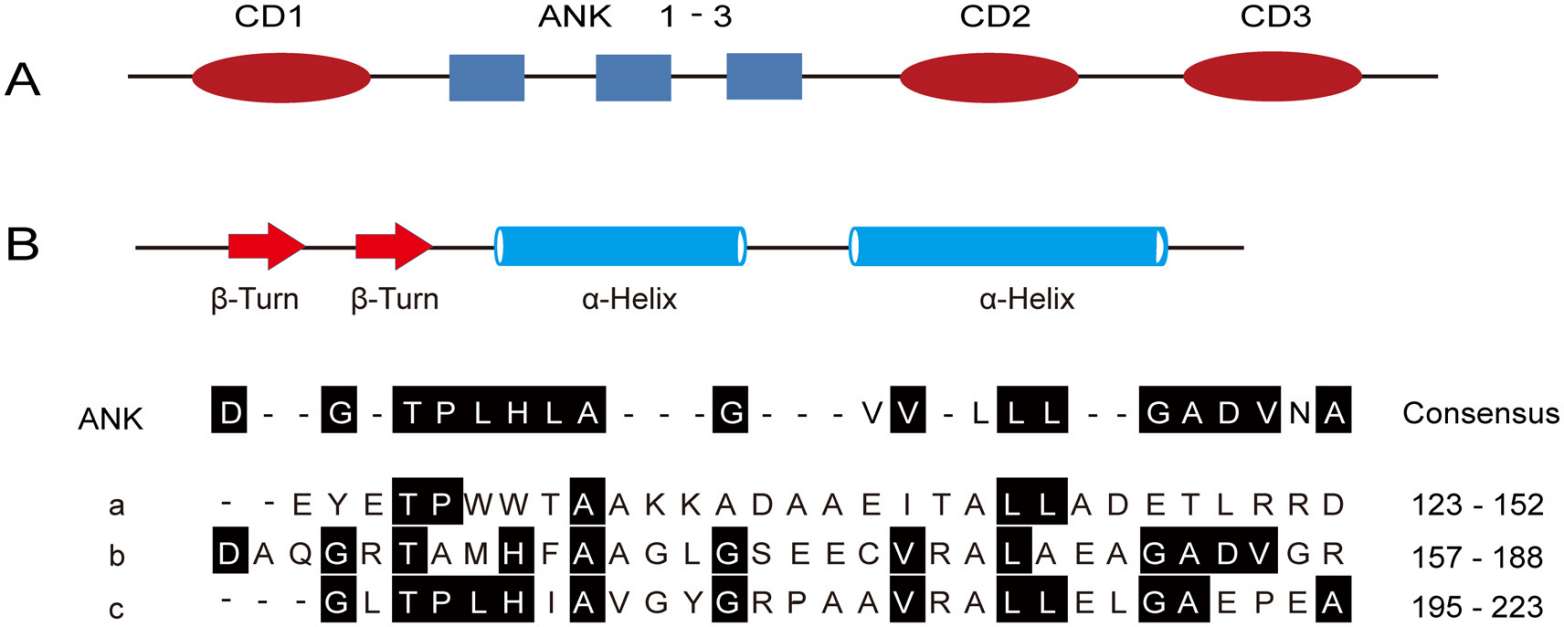
Domain organization of OscpSRP43 and structural details of ANK 1–3. A, OscpSRP43 is composed of three CDs (red ellopses) and three ankyrin repeats (grey boxes); B, Structure of ankyrin-repeat domains (ANK1, residues 123–152; ANK2, 157–188; ANK3, 194–223). CD, chromodomain; ANK, anyrin-repeat domain.

Protein analysis using the Pfam database (Pfam.xfam.org) reveals that OscpSRP43 consists of two major domains: ankyrin-repeat domain and chromodomain (Fig 5A). The ankyrin-repeat domain (ANK) contains three sub-domains (ANK1, ANK2 and ANK3) in tandem covering 101 amino acid residues from 123 to 223 with a variable degree of conservation (Fig 5B). The ankyrin-repeat domain was first identified in the yeast cell cycle regulator Swi6/Cdc10 and the *Drosophila* signaling protein Notch [34]. It assembled from a tandem motif of about 33 amino acids and was defined as a β-hairpin-helix-loop-helix (β2α2) structure mediating protein-protein interactions [35].

The chromodomain (CD) of OscpSRP 43 contains three subdomains as well: CD1 is located in the N-terminal in front of ANK1 between residues 66 and 129, CD2 and CD3 are in tandem and located in the C-terminal covering the residues from 269 to 372. The chromodomain was initially identified in two *Drosophila* proteins: Polycomb (PC) and Heterochromatin Protein 1 (HP1) [36]. They mediate protein-protein or protein-nucleic acid interactions [37].

**Fig 6:**
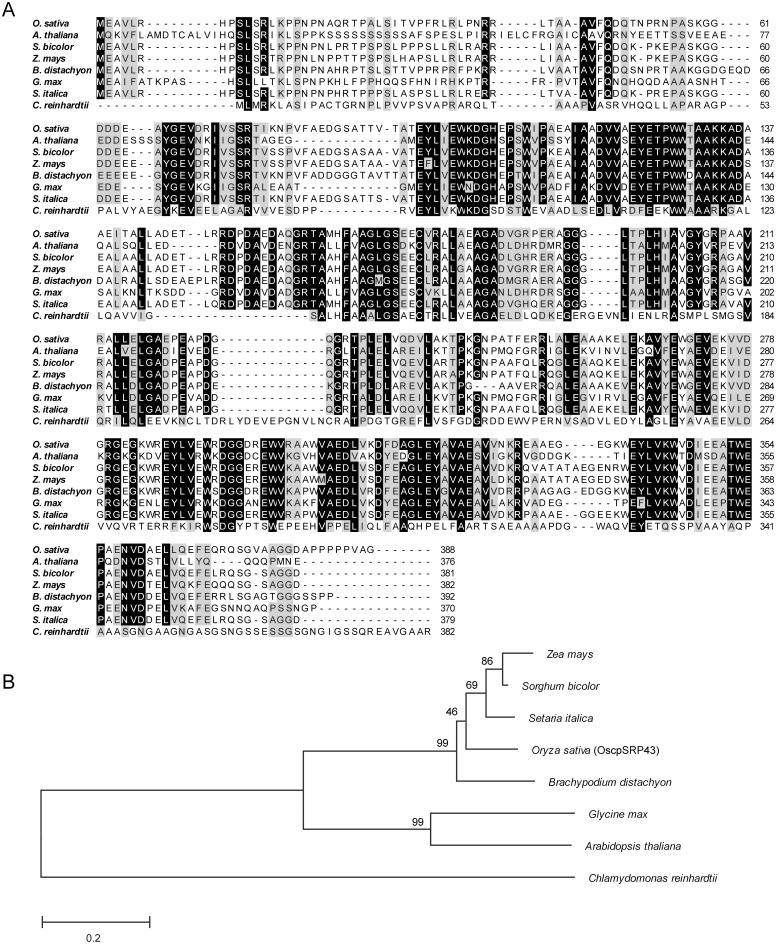
Sequence aligment and phylogenetic analysis of OscpSRP43 homologs. A, Comparison of amino acid sequences of OscpSRP43 homologs. Residues identical to OscpSRP43 are shaded. Accession numbers for the respective protein sequences are as follows: *Zea mays* (NP_001168899); *Sorghum bicolor* (XP_002465920); *Setaria italica* (XP_004985832); *Oryza sativa* (NP_001048866); *Brachypodium distachyon* (XP_003562136); *Glycine max* (XP_003537753); *Arabidopsis thaliana* (NC_003071) *and Chlamydomonas reinhardtii* (AGC59877). B, Dendrogram of OscpSRP43 hommologs. The Neighbor-joining tree using percentage identities was constructed based on a multiple sequence alignment generated with the program MEGA 5.1.

A database search (http://www.ncbi.nlm.nih.gov/blast/) reveals that homologus proteins with OscpSRP43 are presented in *Arabidopsis thaliana*, *Sorghum bicolor*, *Zea mays*, *Glycine max*, *Brachypodium distachyon*, *Setaria italica* and *Chlamydomonas reinhardtii*. The similarity level of cpSRP43 to OscpSRP43 from high to low is *S*. *italica* (83.33%), *S*.*bicolor* (80.87%), *Z*. *mays* (78.89%), *B*. *distachyon* (76.31%) and *G*. *max* (47.70%), *A*.*thaliana* (45.59%) and *C*. *reinhardtii* (21.79%)(Fig 6A). As expected, these homologus can be classified into three groups as the monocot group, dicot group and algae group (Fig 6B). Although OscpSRP43 has a higher similarity to most of the homologs, unfortunately, functions of the other homologs were largely unknown except AtcpSRP43 and CrcpSRP43.

### *OscpSRP43* is constitutively expressed and OscpSRP43 is located in chloroplasts

**Fig 7:**
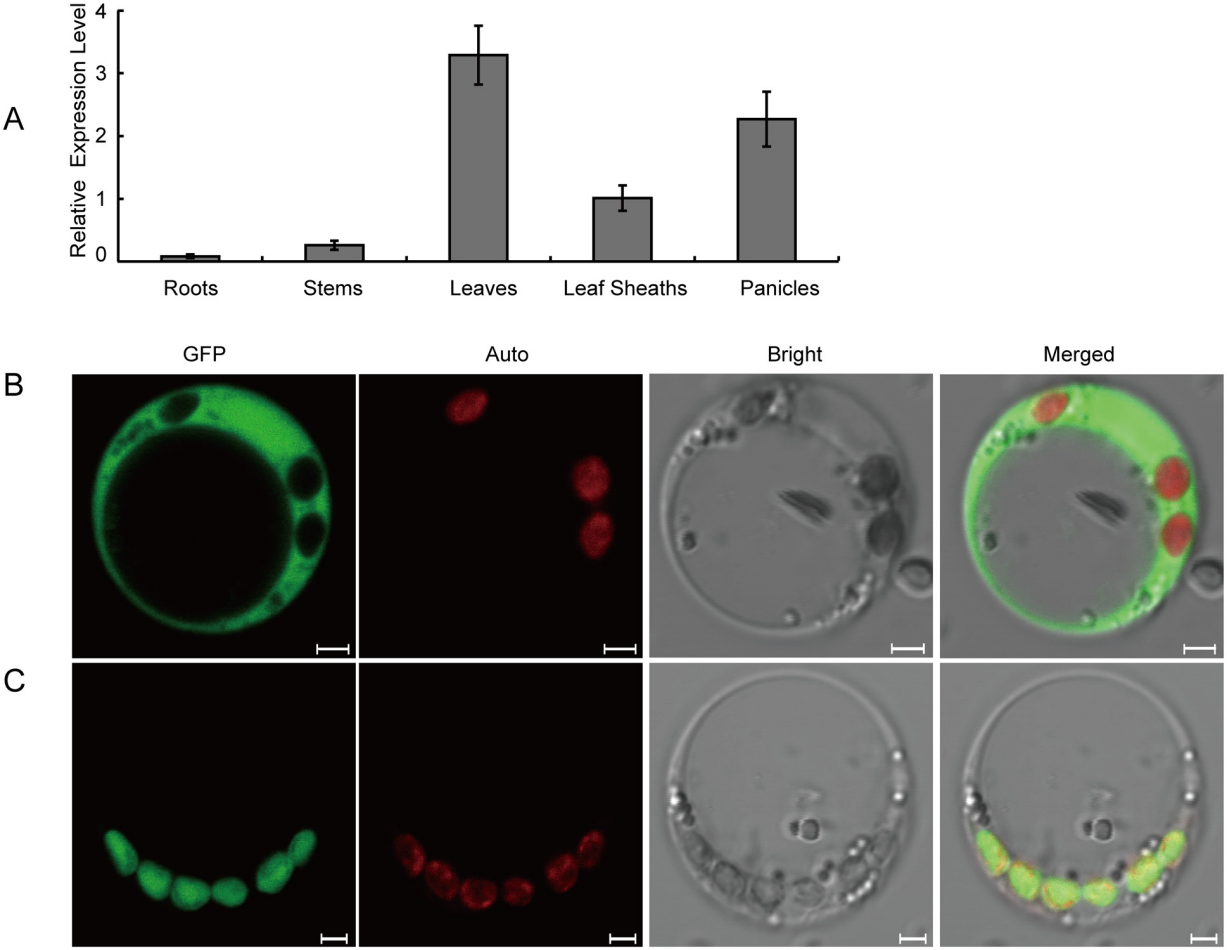
Expression of *OscpSRP43* and subcellular localization of OscpSRP43. A, Relative expression of *OscpSRP43* in different organs of the wild type IR64 at the heading stage; B, Transient expression of GFP protein in rice protoplasts; C, Transient expression of OscpSRP43:GFP fusion protein in rice protoplasts; GFP, green fluorescence protein and OscpSRP43:GFP fusion protein; Auto, Chl auto fluorescence; Bright, bright field; Merged, merged image of GFP, Auto and Bright.

To determine the relative abundance of the transcripts, we performed quantitative reverse transcription-PCR (qRT-PCR) using RNA samples from different parts of IR64 plants. As shown in Fig 7A, *OscpSRP43* was expressed in various organs including the roots, stems, leaves, leaf sheaths and panicles at the heading stage with the highest level of expression in the leaf. The results indicated that *OscpSRP43* was expressed constitutively.

It has been reported that AtcpSRP43 was targeted to the chloroplast [38]. To determine the subcellular localization of OscpSRP43 in rice, we first performed analysis using TargetP (http://www.cbs.dtu.dk/services/TargetP/) and ChloroP (http://www.cbs.dtu.dk/services/ChloroP/) softwares, both results suggested that OscpSRP43 was located in the chloroplast. To determine its actual subcellular localization, we performed a transient expression assay using rice protoplasts. Confocal microscopy observation showed that the free GFP signal was dispersed through the cytoplasm in the rice protoplasts (Fig 7B) whereas the green fluorescent signal of the OscpSRP43:GFP fusion protein was co-localized with the autofluorescent signals of chlorophylls in the chloroplasts (Fig 7C). The results suggested that OscpSRP43 was targeted to the rice chloroplasts.

### Altered expression of genes associated with Chl biosynthesis and photosynthesis

Chl biosynthesis and photosynthesis are tightly regulated by the coordinated expression of plastid and nuclear genes. To investigate the expression changes of genes associated with Chl biosynthesis and photosynthesis in the mutant, we performed quantitative reverse transcription-PCR (qRT-PCR) analysis.

**Fig 8:**
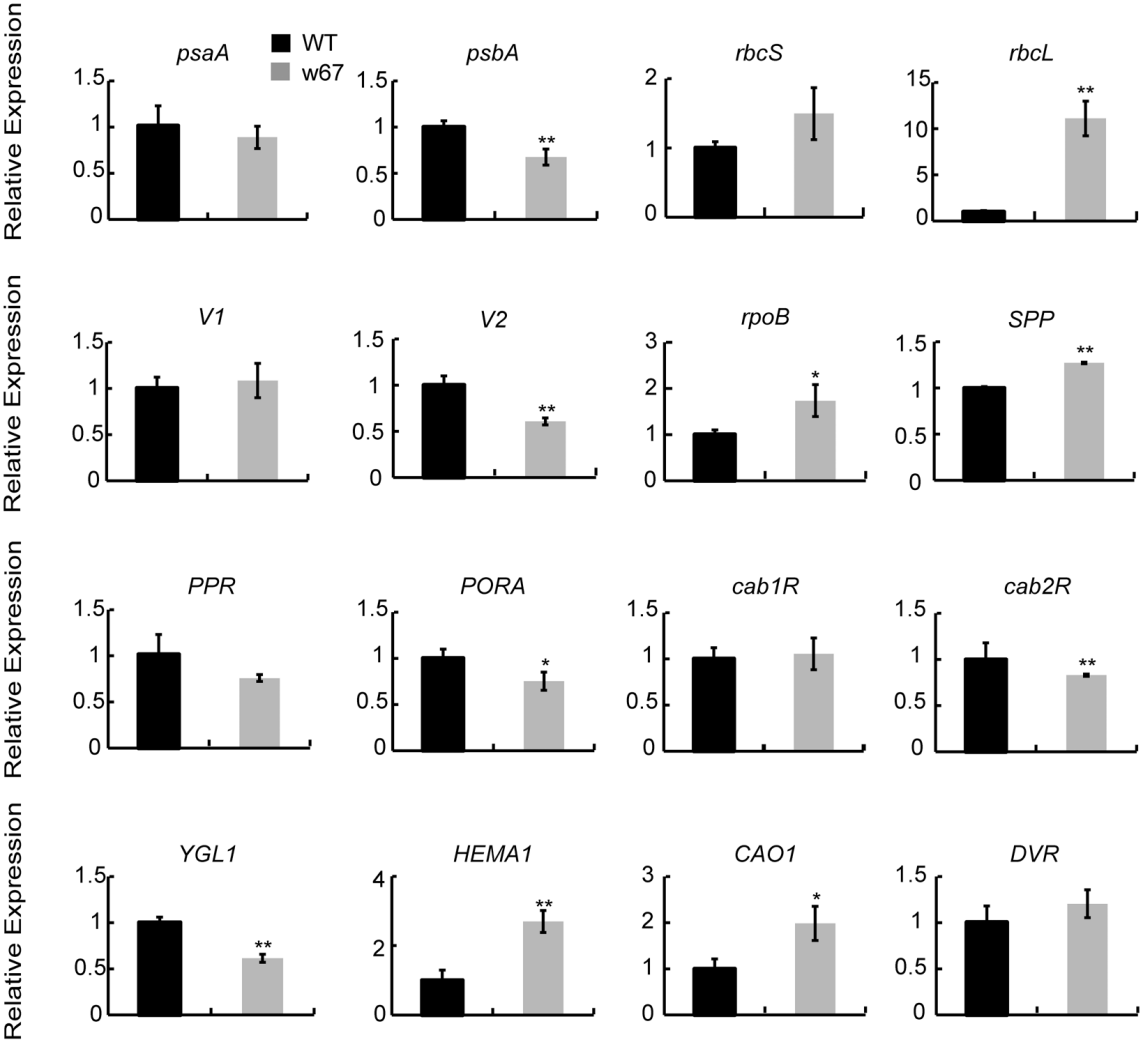
Real-time quantitative reverse transcription PCR analysis. The accession numbers and primer sequences of 16 genes associated with Chl biosynthesis and photosynthesis in IR64 and w67 are listed in S3 Table. The expression levels were determined by real-time PCR and normalized to the *ubiqutin*. The values are presented as the mean ± SD from three biological replicates. *, Significance at P ≤ 0.05; **, Highly significance at P ≤ 0.01.

Compared with the wild type IR64, the expression of *HEMA1* (encoding glutamyl tRNA reductase) and *CAO1* (encoding chlorophyllide a oxygenase) were significantly up-regulated, *PORA* (encoding NADPH-dependent protochlorophyllide oxidoreductase) and *YGL1* (encoding chlorophyll synthase) were significantly down-regulated in the mutant while the expression of *DVR* (encoding 3,8-divinyl protochlorophyllide a 8-vinyl reductase) was similar to the wild type (Fig 8), indicating that the mutation leads to Chl deficiency by affecting the expression of an array of Chl synthetic genes.

The transcript levels of 11 photosynthesis or chloroplast biogenesis-related genes were determined. Three gene including *rbcL*, *rpoB* and *SPP* were significantly up-regulated (Fig 8), three genes including *psbA*, *V2* and *cab2R* were apparently down-regulated in the mutant while the expressions of five genes (*cab1R*, *PPR*, *psaA*, *rbcS* and *v1*) were similar to those of the wild type (Fig 8), indicating that the impaired photosynthesis in the mutant may result from a collaborative effect of a group of genes associated with photosynthesis. Thus, these result indicated that OscpSRP43 played an important role for the normal development of chloroplasts and photosynthesis in rice. Taken together, Loss of function to *OscpSRP43* would lead to a massive disordered expression of the genes associated with chlorophyll metabolism and photosynthesis.

## Discussion

Chlorophyll deficient mutants are ideal materials for understanding of mechanisms involved in chlorophyll metabolism, chloroplast biogenesis and photosynthesis. Up to now, a number of rice mutants with abnormal leaf coloration have been identified and characterized [16, 39, 40]. In this study, we report a novel rice chlorophyll-deficient mutant w67 which exhibits a yellow-green leaf phenotype in the whole life period. w67 is typical Chl-deficient mutant with a low level of Chl and an elevated ratio of the Chl a/b most likely resulted from the impaired development of chloroplasts. Similar phenotypes to w67 have been reported in *Arabidopsis thaliana* and *Chlamydomonas reinhardtii* [38, 41, 42]. Furthermore, w67 shows a lowered performance in major agronomic traits presumably because of the reduced capacity of photosynthesis. Interestingly, 1000-grain weight is not affected probably due to the longer life duration in the mutant.

The map-based cloning and functional complementation assay indicate that the intronless single copy gene *OscpSRP43* is responsible for the yellow-green phenotype. The homolog of *OscpSRP43* in *Arabidopsis* has been identified in the chaos mutant showing deficient in the production of AtcpSRP43 [38]. Similarly to AtcpSRP43, OscpSRP43 is also targeted to the chloroplast in rice. Furthermore, OscpSRP43 shares 45.59% peptide identity with AtcpSRP43. Both proteins are also similar in structure by containing 3–4 tandem ankyrin-repeat domains flanked by three chromodomains, CD1 is located in the N-terminal in front of ANK1 while CD2 and CD3 are located in the C-terminal right after ANK3/ANK4 [43]. This conserved structure may imply that both of them have similar biological function in protein transportation.

The *Arabidopsis* cpSRP43 plays an important role for protein transportation into thylakoids in the chloroplast signal recognition particle (cpSRP) pathway. This pathway is responsible for targeting the D1 protein and light-harvesting chlorophyll-binding proteins (LHCPs) to the thylakoid by a conserved co-translational as well as post-translational transport mechanism [44]. The cpSRP pathway mainly involves three important components: the cpSRP proteins, the membrane-bound SRP receptor cpFtsY, and the integral membrane protein Alb3 [45]. Each component is necessary for the normal growth and development of a plant. Mutations in cpSRP43 or cpSRP54 would lead to pale yellow leaves in *Arabidopsis* while mutations in CrcpSRP43 would result in a low chlorophyll content, high Chl a/b ratio and yellow-green phenotype in *Chlamydomonas reinhardtii* [40,41]. The defects in cpFtsY and Alb3 would result in the death of seedlings [46,47]. During the transportation of a LHCP into the thylakoid, cpSRP43 binds to the L18 motif of a LHCP [43,48] together with cpSRP54 to form a soluble transit complex [49]. Then the receptor cpFtsY recognizes this soluble transit complex and form a membrane-bound complex [50]. Finally, integration of a LHCP into the thylakoid membrane is mediated by the integral membrane protein ALB3 [45]. Therefore, we presumably assume that the defect in OscpSRP43 may completely or partially block the transportation of LHCPs into thylakoids leading to the impaired development of chloroplasts and photosynthesis as well.

The defect of OscpSRP43 seems to affect the expression of a large number of genes associated with Chl metabolism, chloroplast development and photosynthesis. In the present study, the upregulated expression of *OsHEMA1* and *OsCAO1* may be due to a feedback enhancement of expression resulting from the impaired OscpSRP43 function. The deficient in Chl level and yellow-green phenotype in w67 is likely due to the apparent down-regulation of *OsYGL1* and *OsPORA* as both of them are responsible for the biosynthesis of Chl a and Chl b [13]. *Oscab2R* is a LHCP encoding gene and its significant down-regulation is probably because of a feedback inhibition of impaired cpSRP43 function. It is known that the cpSRP43-cpSRP54-LHCP complex is critical to the transportation of LHCPs such as cab2R to thylakoids for a normal function of chloroplast development [51]. Any defect in cpSRP43 would presumably affect LHCPs accumulation and trafficking and lead to the malformation of the light-harvesting complex which consequently results in the impaired development of chloroplasts, chlorophyll metabolism and photosynthesis as well as the chlorophyll-deficient phenotype. However, it is still not clear why *OsrbcL*, *OsrpoB* and *OsSPP* are apparently upregulated and *OspsbA* and *OsV2* are significantly down-regulated. The isolation of *OscpSRP43* in rice and further functional characterization would provide insight into the mechanism of the cpSRP pathway in the monocot model plant.

## Supporting Information

S1 Fig Constructs for complemtary test and subcellular location. A, Complementary vector pCAMBIA1300-w67; B, Subcelluar location vector PAN580-w67.
(TIF)

S1 Table Segregation analysis of F_3_ lines. (DOC)

S2 Table Markers used for mapping. (DOC)

S3 Table List of genes for qRT- PCR analysis. (DOC)
