# Supplementary figures and images for: Oryza sativa Chloroplast Signal Recognition Particle 43 (OscpSRP43) Is Required for Chloroplast Development and Photosynthesis
Source: PLoS One. 2015 Nov 23;10(11):e0143249. doi: 10.1371/journal.pone.0143249 (PMC4657901; doi:10.1371/journal.pone.0143249)

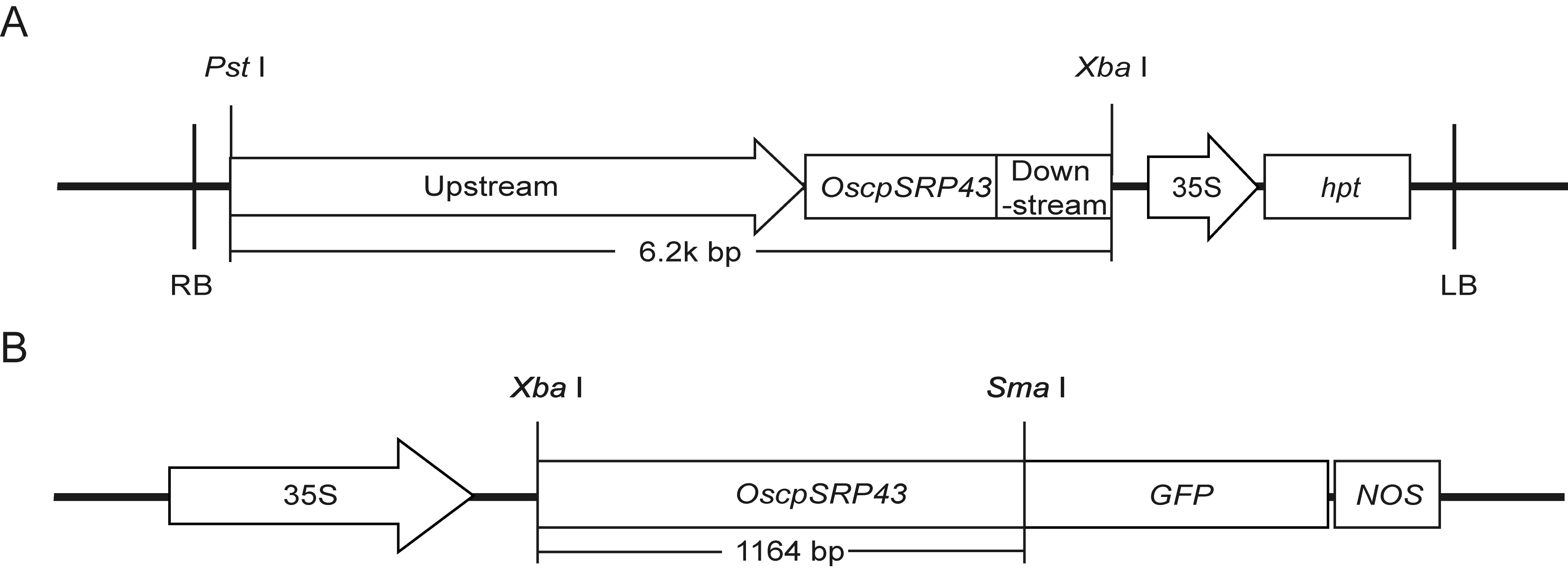

Supplement: S1 Fig — A, Complementary vector pCAMBIA1300-w67; B, Subcelluar location vector PAN580-w67. (TIF) [file pone.0143249.s001.tif]
