# Supplementary material for: Oryza sativa Chloroplast Signal Recognition Particle 43 (OscpSRP43) Is Required for Chloroplast Development and Photosynthesis
Source: PLoS One. 2015 Nov 23;10(11):e0143249. doi: 10.1371/journal.pone.0143249 (PMC4657901; doi:10.1371/journal.pone.0143249)
Supplement: S1 Table — (DOC) [file pone.0143249.s002.doc]

**S1 Table. Segregation analysis of F_3_ lines**

| **Cross** | **Line** | **Total No. plants** | **No. green leaf plants** | **No. yellow-green**  **leaf plants** | **P_(3:1)_** |
| --- | --- | --- | --- | --- | --- |
| w67/02428 | 1 | 261 | 192 | 69 | 0.59 |
|  | 2 | 171 | 134 | 37 | 0.31 |
|  | 3 | 154 | 118 | 36 | 0.64 |
| w67/Moroberekan | 1 | 52 | 38 | 14 | 0.75 |
|  | 2 | 132 | 101 | 31 | 0.69 |
|  | 3 | 212 | 152 | 60 | 0.27 |
