# Supplementary material for: Oryza sativa Chloroplast Signal Recognition Particle 43 (OscpSRP43) Is Required for Chloroplast Development and Photosynthesis
Source: PLoS One. 2015 Nov 23;10(11):e0143249. doi: 10.1371/journal.pone.0143249 (PMC4657901; doi:10.1371/journal.pone.0143249)
Supplement: S2 Table — (DOC) [file pone.0143249.s003.doc]

**S2 Table.** **Markers used for mapping.**

| **Marker** | **Forward primer (5’–3’)** | **Reverse primer (5’-3’)** | **Product size (bp)** |
| --- | --- | --- | --- |
| ID32 | CACATGATCCGAGACATGAATAA | GCTGCAATCCTAGGTAGCTAACA | 206 |
| ID9 | TTACAGTTTAGACTAAAGCCTT | ATTCCTCATTGTTAGCAGTA | 213 |
| RM14288 | GTACCGCAGCTTGATCCCTAGC | CCCAAATAGCGTGACAAAGTGC | 187 |
| RM14302 | CAACCGATCCCAGAGATCAAGG | GCAAGAGAGGGAAGAGGAAGAGG | 78 |
| RM14341 | GCAGATCATAATGGTTCAGAGTGC | GTACACGCAGATGGTTGATCG | 121 |
| RM14412 | CTTTGGTGGGTGTTGGGCTTGG | TGGACTTGGCCGCTGTTCTGG | 99 |
| RM321 | CCAGATTATTTCCTGAGGTC | CACTTGCATAGTTCTGCATTG | 191 |
| RM569 | CTGCGTCAGATTTCTCCTCTTCG | ACATTCTCGCTTGCTCCTCTCG | 116 |
| RM5761 | AAGATTACCCGGGATTGTAGTGG | AGTCTAGTCGTCCTCTTCACATGG | 98 |
| RM6013 | CTCGTCTTCTTCACGAAGGTCTTGC | TCTGCGCCGTCTCACTCACG | 131 |
| RM6883 | CCCTGTTGATGGTTGATTTCTCC | CAAGATCAAAGCACATGAGTCTGC | 153 |
