# Supplementary material for: Oryza sativa Chloroplast Signal Recognition Particle 43 (OscpSRP43) Is Required for Chloroplast Development and Photosynthesis
Source: PLoS One. 2015 Nov 23;10(11):e0143249. doi: 10.1371/journal.pone.0143249 (PMC4657901; doi:10.1371/journal.pone.0143249)
Supplement: S3 Table — (DOC) [file pone.0143249.s004.doc]

**S3 Table. List of genes for qRT- PCR analysis.**

| **Gene** | **Forward primer (5’–3’)** | **Reverse primer (5’–3’)** | **GenBank Accession** |
| --- | --- | --- | --- |
| *cab1R* | AGATGGGTTTAGTGCGACGAG | TTTGGGATCGAGGGAGTATTT | X13908 |
| *cab2R* | TGTTCTCCATGTTCGGCTTCT | GCTACGGTCCCCACTTCACT | X13909 |
| *CAO1* | GATCCATACCCGATCGACAT | CGAGAGACATCCGGTAGAGC | J013116K15 |
| *DVR* | CGAGCCCAGGTTCATCAAGGTGC | CCTCCCGATCTTGCCGAACTCC | ADE43128 |
| *HEMA1* | CGCTATTTCTGATGCTATGGGT | TCTTGGGTGATGATTGTTTGG | J013000F15 |
| *PORA* | TGTACTGGAGCTGGAACAACAA | GAGCACAGCAAAATCCTAGACG | AK065236 |
| *PPR1* | CTAAGACCGAATGACAAATGC | GCACTGCCAACAAGAATACC | AY584749 |
| *psaA* | GCGAGCAAATAAAACACCTTTC | GTACCAGCTTAACGTGGGGAG | AAS46121 |
| *psbA* | CCCTCATTAGCAGATTCGTTTT | ATGATTGTATTCCAGGCAGAGC | AAS46104 |
| *­rbcL* | CTTGGCAGCATTCCGAGTAA | ACAACGGGCTCGATGTGATA | AAS46127 |
| *rbcS* | TCCGCTGAGTTTTGGCTATTT | GGACTTGAGCCCTGGAAGG | X07515 |
| *rpoB* | TTTGGTTTCGATGTGCA | TATGGTCTAATTCCGAGCGGT | NP_039373 |
| *SPP* | CGGAGAGGAAACATAATGAC | ATAGGCATTTGTCTTTGTCTC | AK066566 |
| *V1* | TGGAGGTCGGGACAGAGGA | CGAGGAGCACCACCATCAC | AB550822 |
| *V2* | CGACAAGCAGAGCGAAGCG | AGGTTGCTGCTCCTTGAATGT | AB267728 |
| *YGL1* | AACCTTACCGTCCTATTCCTT | CCATACATCTAACAGAGCACCC | EF432576 |
| *OscpSRP43* | CCTTTCCGGCTTCGTCTCC | CCTTGCTCGCTGGGTTTCTT | KT581950 |
| *ubq* | GCTCCGTGGCGGTATCAT | CGGCAGTTGACAGCCCTAG | AF184280 |
